# Draft Genome Sequence of *Tomitella biformata* AHU 1821^T^, Isolated from a Permafrost Ice Wedge in Alaska

**DOI:** 10.1128/genomeA.00066-14

**Published:** 2014-02-13

**Authors:** Kentaro Funo, Wataru Kitagawa, Michiko Tanaka, Teruo Sone, Kozo Asano, Yoichi Kamagata

**Affiliations:** a Graduate School of Agriculture, Hokkaido University, Sapporo, Japan; b Bioproduction Research Institute, National Institute of Advanced Industrial Science and Technology (AIST), Sapporo, Japan

## Abstract

*Tomitella biformata* AHU 1821^T^ was isolated and cultured from a permafrost ice wedge, aged presumably about 25,000 years, in the Fox permafrost tunnel (64.952°N 147.617°W), Alaska. These genome data provide the basis for investigating *T. biformata* AHU 1821^T^, identified as a long-term survivor of the extremely cold and closed environment.

## GENOME ANNOUNCEMENT

Ice wedges are wedge-shaped ancient ice pieces and are among the most common features of permafrost regions in extremely cold, high-latitude areas of the world (1). Although there are a number of reports of bacteria in frozen environments, like permafrost, only a few investigations have ever been made for bacteria within these ice wedges.

We previously reported on the isolation of bacteria that had been preserved within a permafrost ice wedge for ~25,000 years (2). We found a new bacterium, *Tomitella biformata* AHU 1821^T^ (= DSM 45403^T^ = NBRC 106253^T^), from an ice wedge in the Fox permafrost tunnel (64.952°N 147.617°W), Alaska (3). The genus we proposed is within the suborder *Corynebacterineae*, and the strain is a Gram-positive, aerobic, non-spore-forming, psychrophilic bacterium, growing at -5° C to 27° C, at pH 5 to 10 (3).

The strain increased membrane fluidity at subzero temperatures by modulating the fatty acid composition of the cytoplasmic membrane (3, 4). In the oxygen-limited cultures, it enters into the nondividing state, in which it remains alive but loses its ability to grow or divide under standard cultivation conditions (5). The nondividing cells can be resuscitated by its resuscitation-promoting factor (Rpf) (5–7). Due to these remarkable survival capabilities, *T. biformata* AHU 1821^T^ can be considered a potential model organism for better understanding adaptation or survival mechanisms under extreme conditions.

The genome of *T. biformata* AHU 1821^T^ was determined using the Illumina HiSeq 2000 paired-end technology provided by the Hokkaido System Science Co., Ltd. (Sapporo, Hokkaido, Japan). This sequencing run yielded 19,282,552 high-quality filtered reads with 101-bp paired-end sequencing, providing approximately 410× genome coverage. The genome was assembled using the Velvet assembler version 1.1.02 (hash length, 75 bp) (8). The final assembly contained 113 contigs of total size 4,709,534 bp, with 69.3% G+C content and an N_50_ length of 230,132 bp. The prediction of protein-coding sequences (CDSs) and annotation were performed by the Microbial Genome Annotation Pipeline (http://www.migap.org/), which utilizes MetaGeneAnnotator (9), RNAmmer (10), tRNAScan-SE (11), and BLAST (12). The draft genome sequence of *T. biformata* contains 4,464 putative CDSs and 50 tRNAs. The annotated genome sequences revealed another six *rpf*-related genes in addition to an *rpf* gene characterized previously (5). Furthermore, several genes potentially having a role related to cold adaptation were detected, such as genes for chaperonin GroEL, chaperonin GroES, RecA protein, and cold shock-like proteins. This is the first draft genome sequence for the genus *Tomitella* and also the first report on an isolate from an ice wedge sample. The draft genome of this cold-adapted bacterium not only provides novel information about adaptation or survival mechanisms under extreme conditions but provides a template for many further phylogenetic, comparative genomic, metagenomic, and functional studies.

### Nucleotide sequence accession numbers.

The draft genome sequence has been deposited at DDBJ/EMBL/GenBank under the accession no. BAVQ00000000. The version described in this paper is the first version, BAVQ01000000.
